# Author Correction: The preponderance of nonsynonymous A-to-I RNA editing in coleoids is nonadaptive

**DOI:** 10.1038/s41467-021-23830-5

**Published:** 2021-07-01

**Authors:** Daohan Jiang, Jianzhi Zhang

**Affiliations:** Department of Ecology and Evolutionary Biology, University of Michigan, Ann Arbor, MI USA

**Keywords:** Molecular evolution, Transcriptomics

Correction to: *Nature Communications* 10.1038/s41467-019-13275-2, published online 27 November 2019.

The original version of this Article inadvertently incorrectly used third-party data which represented cDNA sequences rather than the corresponding genomic DNA sequences. In the corrected version these data have been replaced and reanalyzed. Additionally, a coding error was identified and a different alignment tool needed to be used. The following changes have been made:

In the original version of this article, in the ‘Results’ subsection entitled ‘Patterns of restorative and diversifying editing’, the original second paragraph incorrectly stated ‘We identified 3587 one-to-one orthologous genes’. This figure of 3587 was also stated in the second and third paragraphs in the ‘Methods’. The correct version replaces 3587 with 3979 throughout.

The original second paragraph incorrectly stated ‘Of the two categories of nonsynonymous editing sites, the number of diversifying editing sites is 8.4–13.9 times that of restorative editing sites…’. The correct version replaces 8.4–13.9 with 15.7–20.4.

The third paragraph of this section began ‘In each of the four coleoids, *F*_R_ and *L*_R_ are significantly greater than *F*_S_ (Fig. 2b) and *L*_S_ (Fig. 2c), respectively. By contrast, *F*_D_ is significantly smaller than *F*_S_ (Fig. 2b), whereas *L*_D_ is not significantly different from *L*_S_ (Fig. 2c). These results confirm all four predictions of the nonadaptive hypothesis…’. This has been replaced with ‘In each of the four coleoids, *F*_R_ is significantly lower than *F*_S_ when all editing sites are considered (Fig. 2b). However, because the harm-permitting hypothesis concerns relatively highly edited sites, we analyzed sites with editing levels exceeding 10% and found *F*_R_ to be significantly higher than *F*_S_ (Fig. 2b inset). We observed that *L*_R_ is significantly greater than *L*_S_ in all four coleoids (Fig. 2c). By contrast, *F*_D_ is significantly smaller than *F*_S_ when we considered all editing sites (Fig. 2b) or sites with >10% editing levels (Fig. 2b inset). *L*_D_ is not significantly different from *L*_S_ except in the squid (Fig. 2c). These results largely confirm all four predictions of the nonadaptive hypothesis…’. The original version of this paragraph also incorrectly stated ‘For example, in the squid, 33.37% and 13.31% of restorative editing sites but only 22.97% and 6.74% of synonymous editing sites have editing levels >5% and >20%, respectively’. In the correct version, this has been replaced with ‘For example, in the squid, 29.14% and 15.65% of restorative editing sites but only 23.03% and 6.82% of synonymous editing sites have editing levels >5% and >20%, respectively’.

The fourth paragraph of this section incorrectly stated ‘*F*_R_ > *F*_S_ and *F*_D_ < *F*_S_ hold across tissues, but editing level comparisons are mostly nonsignificant, likely due to the reduced statistical power as a result of decreased sample sizes (Supplementary Table 2).’ This has been replaced with ‘*F*_D_ < *F*_S_ holds across tissues, but editing level comparisons as well as comparisons between *F*_R_ and *F*_S_ are mostly non-significant, likely due to the reduced statistical power as a result of decreased sample sizes (Supplementary Table 2)’. Additionally, in the final sentence of the paragraph, ‘*L*_D_ < *L*_S_’ has been changed to ‘*L*_D_ ~ *L*_S_’.

The fourth paragraph also originally stated ‘Both *F*_R_/*F*_S_ and *F*_D_/*F*_S_ generally increase with the editing level. Although *F*_R_/*F*_S_ almost always exceeds 1, *F*_D_/*F*_S_ is smaller than 1, except when the editing level exceeds 60%’ This has been replaced with ‘While both *F*_R_/*F*_S_ and *F*_D_/*F*_S_ generally increase with the editing level, *F*_D_/*F*_S_ is smaller than 1 except when the editing level exceeds 40%’.

In the subsection of the results entitled ‘Accelerated nonsynonymous G-to-A substitutions’, the original second paragraph stated ‘By respectively bootstrapping the two groups of genes 200 times, we found that the above difference is statistically significant (*P* = 0.015)’. In the correct version, (*P* = 0.015) has been replaced with (*P* < 0.005).

In the subsection of the ‘Results’ entitled ‘The potential benefit of shared editing among species’, the original second paragraph stated ‘For editing sites shared between the octopus and bimac, and those shared between the squid and cuttlefish, *F*_R_ and *F*_D_ are both significantly smaller than *F*_S_ (Fig. 4a). By contrast, *L*_R_ and *L*_D_ are both significantly greater than *L*_S_ (Fig. 4b). For the subset of the above shared editing sites that are shared by all four coleoids, *F*_D_ and *L*_D_ are respectively significantly greater than *F*_S_ (Fig. 4a) and *L*_S_ (Fig. 4b), so are *F*_R_ (Fig. 4a) and *L*_R_ (Fig. 4b)’. This has been replaced with ‘For each of the three groups of editing respectively shared between octopus and bimac, between squid and cuttlefish, and among the four coleoids, both *F*_R_ and *F*_D_ are lower than *F*_S_ (Fig. 4a). However, when the editing level exceeds 10%, *F*_R_ is significantly higher than *F*_S_ for shared editing between octopus and bimac and that between squid and cuttlefish, and *F*_D_ is significantly higher than *F*_S_ for shared editing between squid and cuttlefish (Fig. 4a inset). *L*_R_ and *L*_D_ are both higher than *L*_S_ for each group of shared editing (Fig. 4b)’.

The final sentences of this paragraph stated ‘Hence, nonsynonymous editing shared by all four coleoids show strong and consistent adaptive signals, suggesting that a large fraction is adaptive. In comparison, nonsynonymous editing shared between the octopus and bimac, and that shared between the squid and cuttlefish exhibit some but not all signs of adaptation, and the adaptive signals are much weaker, suggesting that only a smaller fraction is adaptive’. This has been replaced with ‘Hence, diversifying editing shared by different coleoids shows adaptive signals, suggesting that a fraction is adaptive’.

The third paragraph incorrectly stated ‘We estimated that, of species-specific diversifying editing sites, 0.47%, 0.52%, 1.12%, and 0.40% are adaptive in the octopus, bimac, squid, and cuttlefish, respectively (see Methods). Similarly, 1.65%, 1.42%, 8.31%, and 4.95% of shared diversifying editing sites are adaptive in the four coleoids, respectively. Taken together, 0.75%, 0.98%, 1.90%, and 1.00% of diversifying editing sites are adaptive in the four coleoids, respectively.’ This has been replaced with ‘We estimated that, of species-specific diversifying editing sites, 0.15%, 0%, 1.20%, and 0.16% are adaptive in the octopus, bimac, squid, and cuttlefish, respectively (see Methods). Similarly, 3.50%, 3.00%, 14.98%, and 12.26% of shared diversifying editing sites are adaptive in octopus, bimac, squid, and cuttlefish, respectively. Taken together, 1.37%, 1.72%, 4.91%, and 2.18% of diversifying editing sites are adaptive in the four coleoids, respectively’.

The original final paragraph of this subsection stated ‘Interestingly, the frequency of such replacements for nonsynonymous editing is significantly greater than that for synonymous editing in a two-tailed Fisher’s exact test (Fig. 4c and Supplementary Table 4). Because it is the shared diversifying editing for which the nature of the benefit is in question, we restricted the analysis to diversifying editing only, but obtained a similar result (Fig. 4c and Supplementary Table 4). It is noteworthy that no synonymous or nonsynonymous editing was found to be replaced with an A-to-C/T substitution among this set of sites (Supplementary Table 4). Our finding suggests that, if anything, nonsynonymous editing is more likely to be replaced with an A-to-G substitution than is synonymous editing, probably because having a genomic G is superior to having a genomic A that cannot be edited to G in all mRNA molecules. In other words, our results reject the first hypothesis and suggest that the nature of the benefit of adaptive A-to-G editing is similar to that of the same nucleotide substitution, although the size of benefit from the former is smaller than that from the latter. Furthermore, the finding in Fig. 4c suggests that the significantly greater *F*_D_ than *F*_S_ for editing shared among all four coleoids is better explained by positive selection promoting the initial fixation of mutations that led to beneficial nonsynonymous editing than purifying selection preventing the loss of beneficial nonsynonymous editing’. This has been changed to ‘Our analysis showed that the frequency of A-to-G substitutions at nonsynonymous editing sites is significantly lower than that at synonymous editing sites (Fig. 4c; Supplementary Table 4). Because it is the shared diversifying editing for which the nature of the benefit is in question, we restricted the analysis to diversifying editing only, but obtained a similar result (Fig. 4C; Supplementary Table 4). Furthermore, A sites edited in at least three coleoid species are more likely to be edited in all four coleoids when the editing is nonsynonymous than when it is synonymous (36.6% vs. 27.6%, *P* = 0.001, chi-squared test; Supplementary Table 4), suggesting that shared nonsynonymous editing is less likely to be lost than shared synonymous editing. Together, these observations suggest that the benefit of adaptive shared editing is the provision of two protein isoforms per edited site in an organism’.

The fifth paragraph of the ‘Discussion' incorrectly stated ‘Our additional analysis suggests that the benefit of these adaptive editing events does not lie in the protein diversity brought by editing, but lies in the superiority of the edited isoform to the unedited version. Furthermore, nonsynonymous editing is more likely than synonymous editing to be replaced with an A-to-G substitution, suggesting that the nature of the benefit of adaptive editing is similar to the corresponding nucleotide substitution but the extent of the benefit is smaller than that of the substitution. Thus, even when RNA editing is advantageous, the advantage does not rely on its characteristic of generating protein diversity; rather, editing appears to be a temporary solution that is eventually replaced by the more advantageous A-to-G substitution. This result contrasts the prevailing view about how coding RNA editing may be adaptive and further argues that coding sequence editing is unlikely the primary function of RNA editing’. This has been changed to ‘Our additional analysis suggests that the benefit of these adaptive editing events lies in the protein diversity brought by editing. While this finding supports the prevailing view on why coding RNA editing may be adaptive, it is important to stress that, based on our estimation, only about 2.5% of nonsynonymous editing appears adaptive in an average coleoid’.

The sixth paragraph of the ‘Discussion’ incorrectly stated ‘Contrary to this interpretation, nonsynonymous editing of the common ancestor of coleoids is more likely than synonymous editing to be replaced with an A-to-G substitution. That is, an A-to-G substitution is preferred over A-to-G editing even when the editing is beneficial. We believe that the observation prompting Liscovitch-Brauer et al.’s erroneous conclusion is caused by an ascertainment bias’. This has been changed to ‘However, we believe that the observation prompting such a conclusion is caused by an ascertainment bias’.

The first paragraph of the ‘Methods’ stated ‘We extracted coding sequences from the previously assembled transcriptomes^27^ on the basis of the annotations in the dataset’. The correct version adds ‘and converted Gs in the sequences to As at edited sites’ after ‘dataset’.

The second paragraph of the ‘Methods’ stated ‘We first made a protein sequence alignment of orthologous sequences using Clustal Omega^63^ and then generated a coding sequence alignment of these genes using PAL2NAL^64^’. The correct version replaces ‘Clustal Omega’ with ‘MAFFT’. Reference 63, ‘Sievers, F. et al. Fast, scalable generation of high-quality protein multiple sequence alignments using Clustal Omega. Mol. Syst. Biol. 7, 539 (2011)’ has been replaced with ‘Katoh, K. & Standley, D. M. MAFFT multiple sequence alignment software version 7: improvements in performance and usability. *Mol. Biol. Evol.* **30**, 772–780 (2013)’ accordingly.

This paragraph also stated ‘Ancestral sequences were inferred using the codeml program in PAML4 ^65^ under default parameters, and the best joint inferences of all interior nodes were used in subsequence analyses.’ The correct version adds ‘without excluding gap sites’ after ‘parameters’.

The original version of this Article also contained errors in Figs. 2b, c, 3 and 4. The correct version of Fig. 2 is:
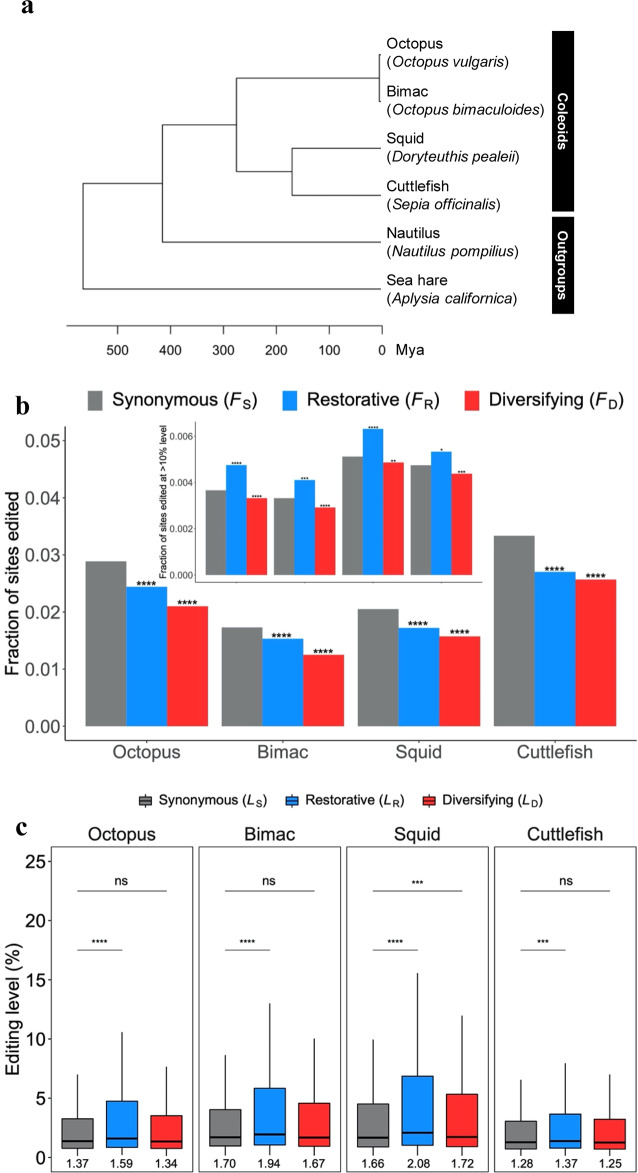


which replaces the previous incorrect version: 
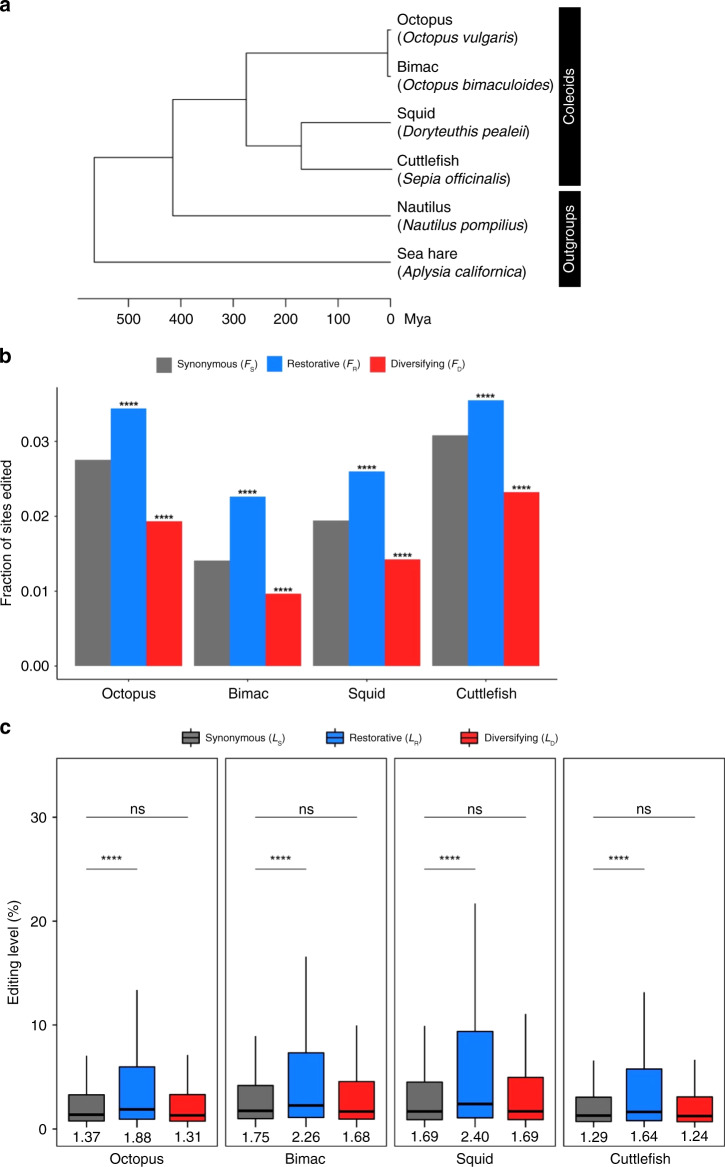


The correct version of Fig. 3 is:
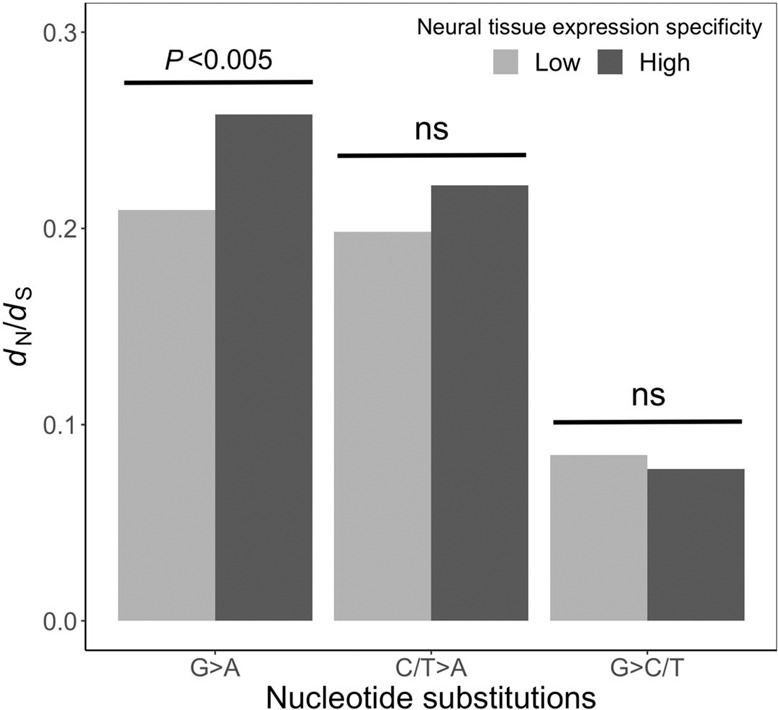


which replaces the previous incorrect version: 
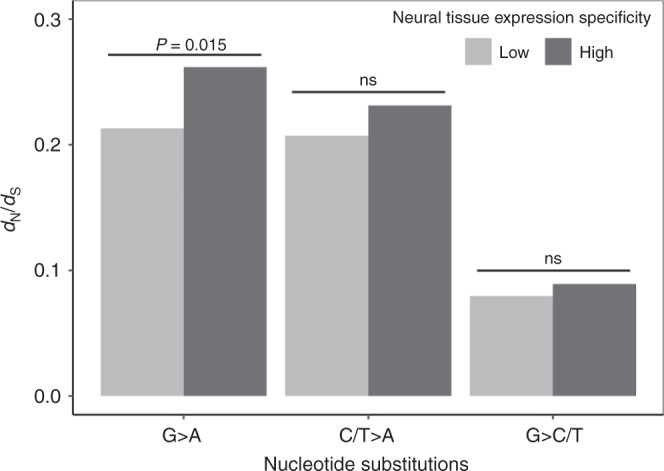


The correct version of Fig. 4 is:
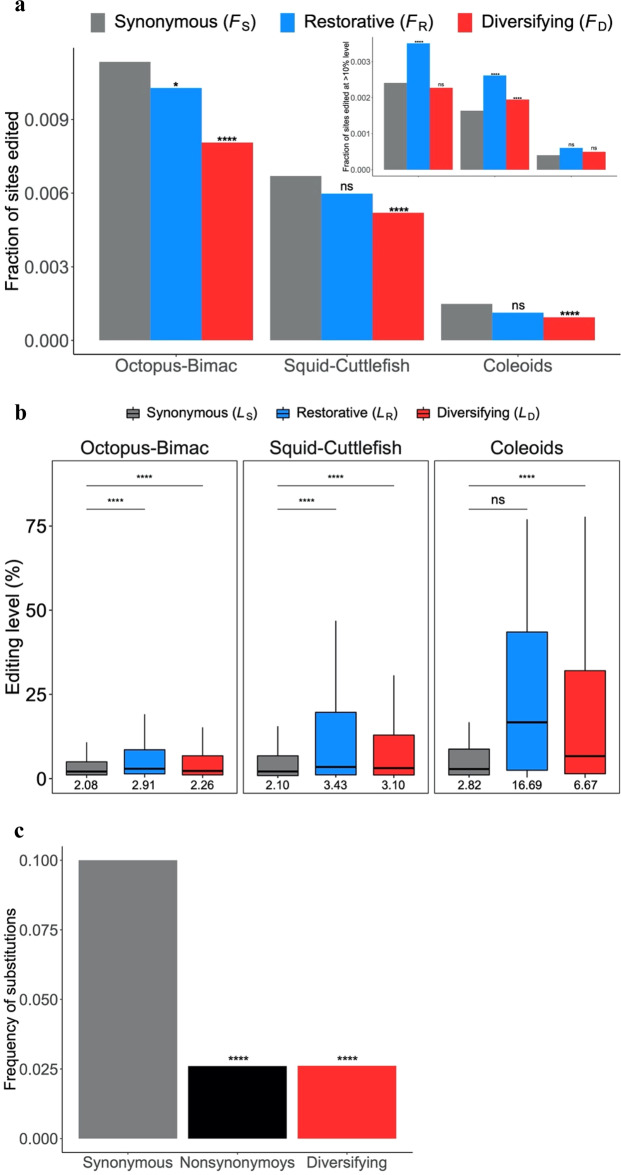


which replaces the previous incorrect version: 
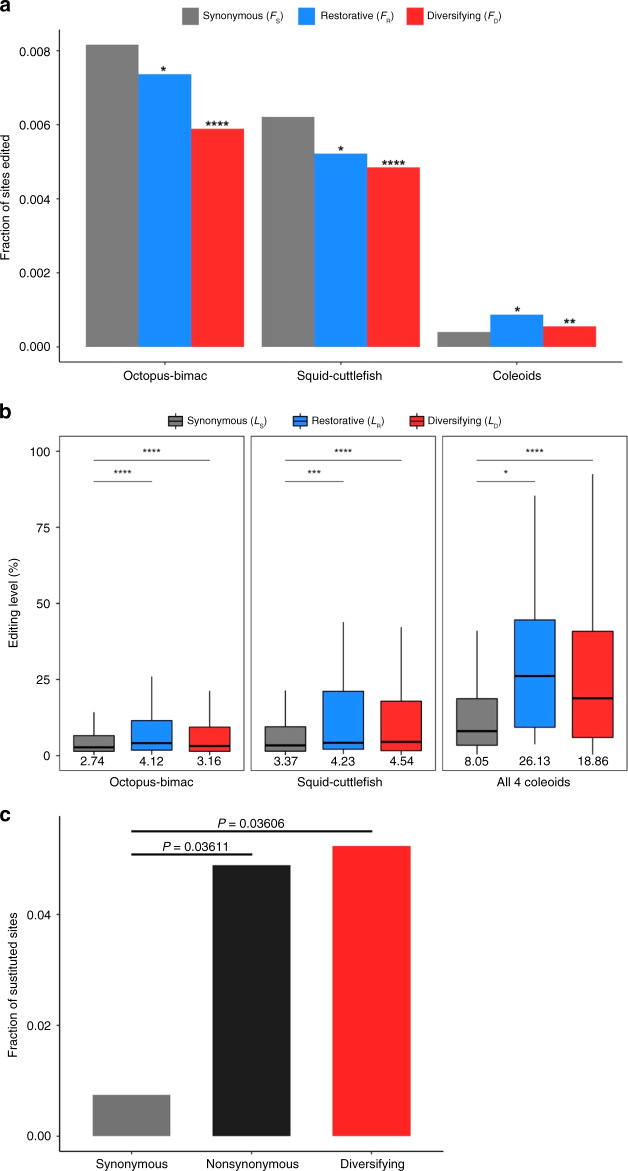


The original captions for Figs. 2b and 4a omitted the following text ‘The inset shows the corresponding fractions of sites with editing levels >10%’. This has been added to the end of the captions for both figures.

The original caption for Fig. 4c incorrectly stated ‘Fraction of sites edited in the common ancestor of the four coleoids that have a genomic G in a coleoid. *P*-values are based on two-tailed Fisher’s exact test’. In the correct version, it states ‘*P*-values are based on two-tailed Fisher’s exact test’ has been replaced with ‘(****, *P* < 0.0001; chi-squared test)’.

These have been corrected in both the PDF and HTML versions of the Article.

The original version of the Supplementary Information associated with this Article contained errors in Supplementary Figs. 1–3 and Supplementary Tables 1–4. The HTML has been updated to include a corrected version of the Supplementary Information; the original incorrect versions of these figures can be found as Supplementary Information associated with this Correction.

The original version of the Source Data associated with this Article contained errors reflecting the changes made to the data and figures above. The HTML has been updated to include a corrected version of the Source Data; the original incorrect version can be found associated with this Correction.

## Supplementary information

Supplementary Materials

Supplementary Information

