# Supplementary material for: The preponderance of nonsynonymous A-to-I RNA editing in coleoids is nonadaptive
Source: Nat Commun. 2019 Nov 27;10:5411. doi: 10.1038/s41467-019-13275-2 (PMC6881472; doi:10.1038/s41467-019-13275-2)
Supplement: Supplementary file 1 — Supplementary Information [file 41467_2019_13275_MOESM1_ESM.pdf]

Supplementary Materials for

"The preponderance of nonsynonymous A-to-I RNA editing in coleoids is nonadaptive"

D. Jiang & J. Zhang (jianzhi@umich.edu)

a

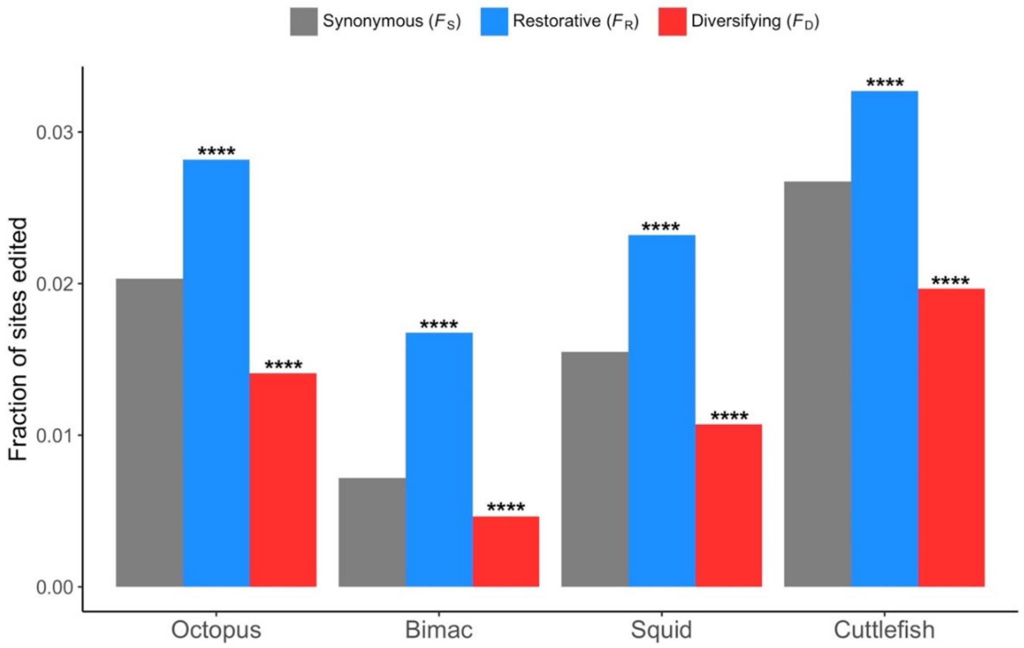

b

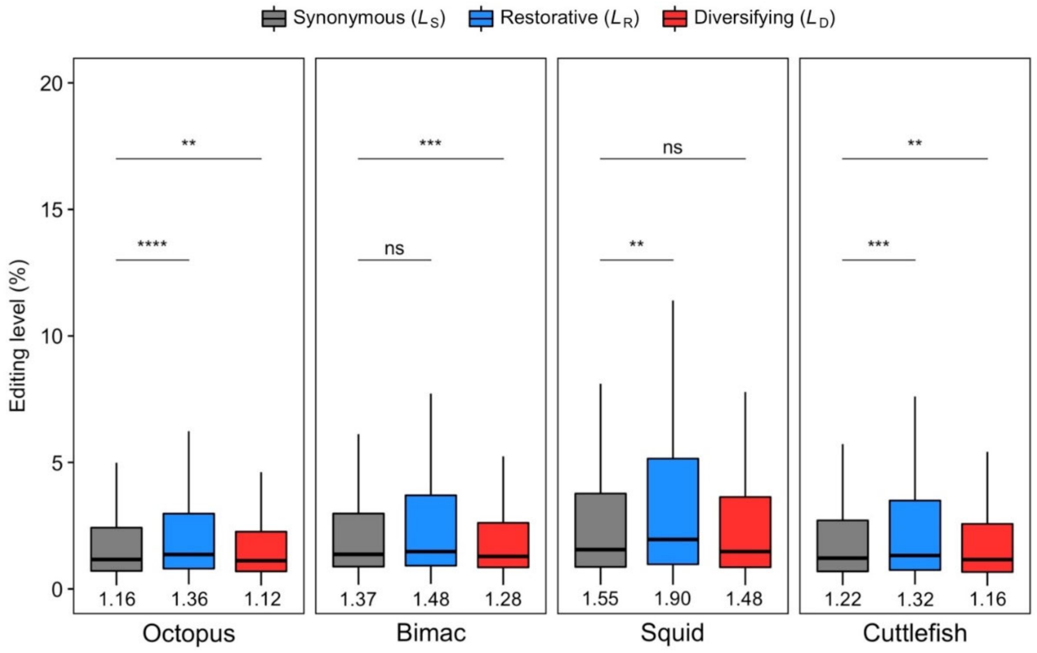

Supplementary Fig. 1. Respectively comparing restorative and diversifying editing with synonymous editing for species-specific editing supports the nonadaptive hypothesis of the preponderance of nonsynonymous editing in coleoids. (**a**) Frequencies of sites with synonymous (F_S), restorative (F_R), and diversifying (F_D) editing, respectively, in each of the four coleoids, for species-specific editing. Significant difference between F_S and F_R (or F_D) are indicated by stars above the bin of F_R (or F_D) (*, P<0.05; **, P<0.01; ***, P<0.001; ****, P<0.0001; ns, not significant; chi-squared test). (**b**) Synonymous, restorative, and diversifying editing levels in

each of the four coleoids for species-specific editing. The lower and upper edges of a box represent the first (qu_1) and third quartiles (qu_3), respectively, the horizontal line inside the box indicates the median (md), and the whiskers extend to the most extreme values inside inner fences, md±1.5(qu_3-qu_1). The median editing levels are also given below the corresponding boxes. Significant differences between L_S and L_R (or L_D) are indicated by stars (*, P<0.05; **, P<0.01; ***, P<0.001; ****, P<0.0001; ns, not significant; Mann-Whitney U test). Note that some identified species-specific editing may in fact be shared among species because of the low detectability of editing caused by low sequencing coverage and/or low editing level. While the group of identified species-specific editing may be contaminated with some shared editing, the group of identified shared editing is not contaminated with species-specific editing. Hence, true signals from species-specific editing can be inferred by comparing between the results here and those in Fig. 4. Specifically, the true signals from species-specific editing after the removal of the contamination from shared editing are inferred as follows. First, because shared editing between two species shows $F_R < F_S$ (Fig. 4a), removal of contamination from shared editing would strengthen the signal of $F_R > F_S$ observed in species-specific editing. In other words, the true signal of $F_R > F_S$ should be even stronger than what is shown here. Second, the true signal of $F_D < F_S$ should be similar to that seen here, because the relationship between F_D and F_S observed here is similar to that in Fig. 4a. Third, the true signal of $L_R > L_S$ should be similar to that seen here, because the relationship between L_R and L_S observed here is similar to that in Fig. 4b. Fourth, because shared editing between two species shows $L_D > L_S$ (Fig. 4b), removal of contamination from shared editing would strengthen the signal of $L_D < L_S$ observed in species-specific editing. In other words, the true signal of $L_D < L_S$ should be even stronger than what is shown here. Source data are provided as a Source Data file.

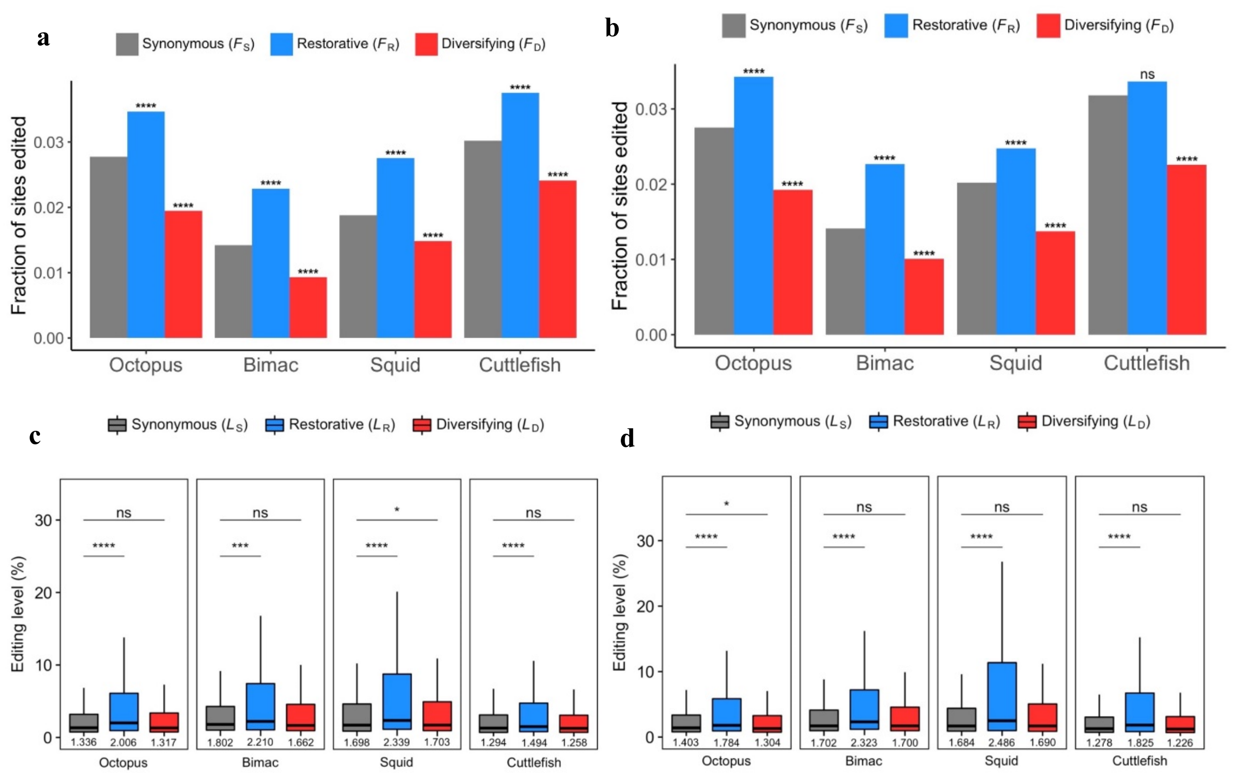

Supplementary Fig. 2. Comparison of editing levels between synonymous and nonsynonymous editing sites of different genes. Genes are ranked by d_N/d_S and are divided into two bins, with bin 1 comprising genes with odd ranks and bin 2 comprising genes with even ranks (see main text for details). (**a**) Comparison between editing frequencies of synonymous editing from bin 1 and those of nonsynonymous editing from bin 2. (**b**) Comparison between editing frequencies of synonymous editing from bin 2 and those of nonsynonymous editing from bin 1. (**c**) Comparison between editing levels of synonymous editing from bin 1 and those of nonsynonymous editing from bin 2. (**d**) Comparison between editing levels of synonymous editing from bin 2 and those of nonsynonymous editing from bin 1. All symbols have the same meanings as in Fig. 2. Source data are provided as a Source Data file.

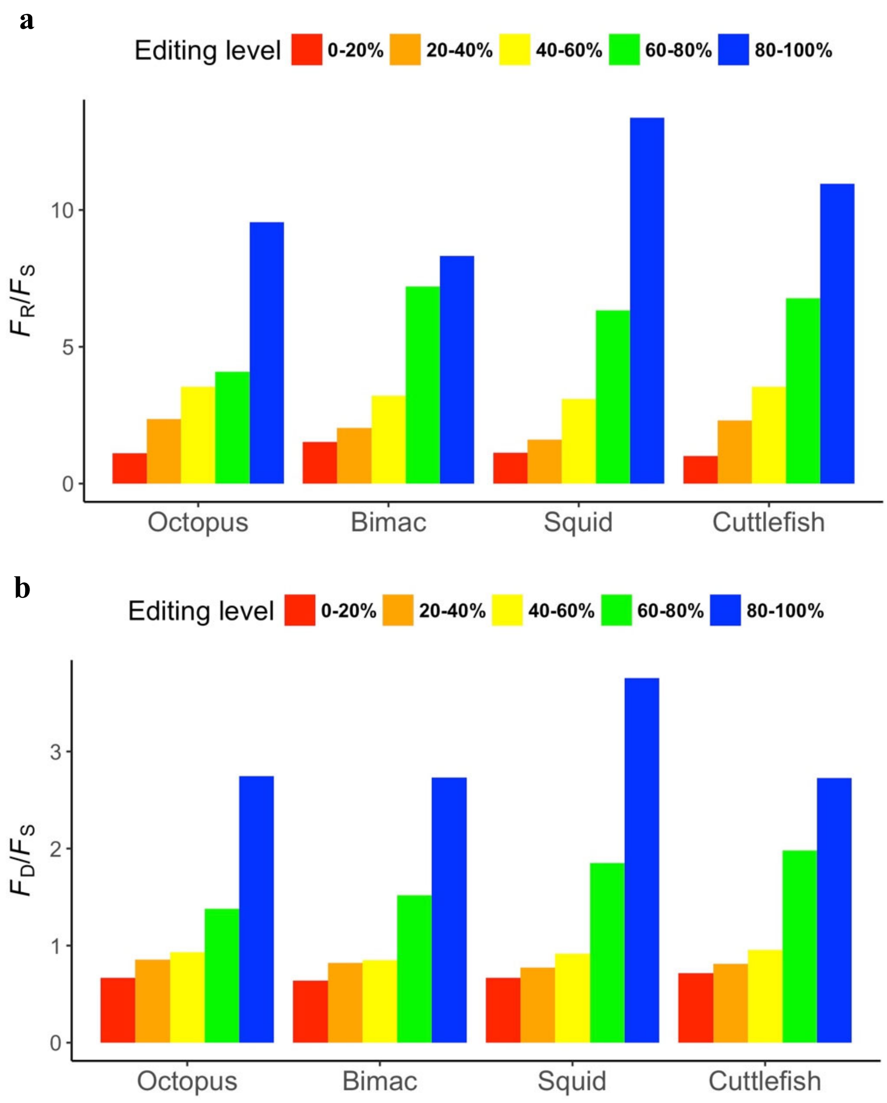

Supplementary Fig. 3. Ratios of editing frequencies of synonymous (F_S), diversifying (F_D), and radical (F_R) editing in various ranges of editing levels. (**a**) F_R/F_S. (**b**) F_D/F_S. Note the different Y-axis scales of the two panels. Source data are provided as a Source Data file.

Supplementary Table 1. Numbers of editing sites of each category. In the parentheses are numbers of species-specific editing.

		Nonsynonymous	
	Synonymous	Restorative	Diversifying
Octopus	19,014 (14,039)	2,818 (2,309)	30,760 (22,422)
Bimac	10,142 (5,167)	1,960 (1,451)	16,013 (7,675)
Squid	13,053 (10,414)	1,838 (1,641)	21,780 (16,412)
Cuttlefish	19,939 (17,300)	2,513 (2,316)	34,906 (29,538)
Shared between octopus and bimac	4,975	509	8,338
Shared between squid and cuttlefish	2,639	197	5,368
Shared among all four coleoids	134	11	376

Supplementary Table 2. Patterns of restorative and diversifying editing in 12 different tissues of the bimac. Stars indicate significant differences from synonymous editing (*, $P < 0.05$; **, $P < 0.01$; ***, $P < 0.001$; ****, $P < 0.0001$; chi-squared test and Mann-Whitney U test are used for comparisons of editing frequencies and editing levels, respectively; #, not significant after the Bonferroni correction for multiple testing). As in the main analysis, editing levels are compared for sites covered by at least 400 RNA-seq reads. No restorative editing site satisfying this condition is found in two tissues; their L_R values are shown as "NA".

**Tissue**	**Editing frequencies**			**Median editing levels (%)**		
	Synonymous (F_S)	Restorative (F_R)	Diversifying (F_D)	Synonymous (L_S)	Restorative (L_R)	Diversifying (L_D)
Axial nerve cord (ANC)	0.0039	0.0072****	0.0027****	1.35	1.88	1.92
Optical lobe (OL)	0.0032	0.0062****	0.0023****	1.95	8.10	1.43
Subesophageal brain (sub)	0.0055	0.0095****	0.0038****	2.32	3.56****	2.60*#
Supraesophageal brain (supra)	0.0053	0.0093****	0.0036****	1.40	2.15****	1.53**#
Posterior salivary gland (PSG)	0.00021	0.00060****	0.00017	0.92	NA	0.16
Skin	0.00034	0.00076****	0.00023****	0.24	0.49	0.23
Sucker	0.00066	0.0015****	0.00051****	0.44	0.39	0.31
Retina	0.00071	0.0018****	0.00051****	0.48	0.37	0.55
Ovary	0.00013	0.00029*#	0.0000084*#	0.39	NA	0.23
Testes	0.00031	0.00066****	0.00022***	0.21	0.22	0.17
Viscera	0.00027	0.00060****	0.00018***	0.19	0.56	0.20
Stage 15 embryo (ST15)	0.0010	0.0023****	0.00074****	0.56	0.53	0.44

Supplementary Table 3. Proportion of editing sites belonging to each editing level range.

Species	Type	0-20%	20-40%	40-60%	60-80%	80-100%
Octopus	Synonymous	95.62%	2.82%	0.95%	0.51%	0.11%
	Restorative	89.78%	5.62%	2.84%	1.75%	0.88%
	Diversifying	93.7%	3.53%	1.29%	1.03%	0.44%
Bimac	Synonymous	94.02%	3.74%	1.58%	0.54%	0.13%
	Restorative	89.62%	4.77%	3.18%	2.43%	0.65%
	Diversifying	91.52%	4.69%	2.04%	1.24%	0.52%
Squid	Synonymous	93.26%	3.98%	1.55%	0.78%	0.42%
	Restorative	86.69%	5.28%	3.96%	4.08%	4.56%
	Diversifying	89.20%	4.43%	2.04%	2.08%	2.24%
Cuttlefish	Synonymous	95.71%	2.73%	1.01%	0.44%	0.11%
	Restorative	88.24%	5.76%	3.29%	2.71%	1.06%
	Diversifying	94.05%	3.04%	1.33%	1.18%	0.39%

Supplementary Table 4. Numbers of sites that are respectively edited, unedited, and substituted in the fourth coleoid among those sites that are edited in the other three coleoids examined.

	Edited A	Unedited A	A-to-G substitution	A-to-C or A-to-T substitution
Synonymous editing	134	0	1	0
Nonsynonymous editing	409	0	20	0
Diversifying editing	382	0	20	0
